# A randomized, double-blind, placebo-controlled clinical trial to evaluate the efficacy and safety of neramexane in patients with moderate to severe subjective tinnitus

**DOI:** 10.1186/1472-6815-11-1

**Published:** 2011-01-11

**Authors:** Markus Suckfüll, Michael Althaus, Barbara Ellers-Lenz, Alexander Gebauer, Roman Görtelmeyer, Pawel J Jastreboff, Hans J Moebius, Tanja Rosenberg, Hermann Russ, Yvonne Wirth, Hagen Krueger

**Affiliations:** 1 University of Munich, Department of Oto-Rhino-Laryngolgy, Marchioninistraße 15, 81377 Munich, Germany; 2 Merz Pharmaceuticals, Frankfurt, Germany; 3 Emory University, Tinnitus and Hyperacusis Center Atlanta, USA

## Abstract

**Background:**

Neramexane is a new substance that exhibits antagonistic properties at α_9_α_10 _cholinergic nicotinic receptors and *N*-methyl-D-aspartate receptors, suggesting potential efficacy in the treatment of tinnitus.

**Methods:**

A total of 431 outpatients with moderate to severe subjective tinnitus (onset 3-18 months before screening) were assigned randomly to receive either placebo or neramexane mesylate (25 mg/day, 50 mg/day and 75 mg/day) for 16 weeks, with assessment at 4-week intervals. The primary (intention-to-treat) efficacy analysis was based on the change from baseline in Week 16 in the total score of the adapted German short version of the validated Tinnitus Handicap Inventory questionnaire (THI-12).

**Results:**

Compared with placebo, the largest improvement was achieved in the 50 mg/d neramexane group, followed by the 75 mg/d neramexane group. This treatment difference did not reach statistical significance at the pre-defined endpoint in Week 16 (*p *= 0.098 for 50 mg/d; *p *= 0.289 for 75 mg/d neramexane), but consistent numerical superiority of both neramexane groups compared with placebo was observed. Four weeks after the end of treatment, THI-12 scores in the 50 mg/d group were significantly better than those of the controls. Secondary efficacy variables supported this trend, with *p *values of < 0.05 for the 50 mg/d neramexane group associated with the functional-communicational subscores of the THI-12 and the assessments of tinnitus annoyance and tinnitus impact on life as measured on an 11-point Likert-like scale. No relevant changes were observed for puretone threshold, for tinnitus pitch and loudness match, or for minimum masking levels. The 25 mg/d neramexane group did not differ from placebo. Neramexane was generally well tolerated and had no relevant influence on laboratory values, electrocardiography and vital signs. Dizziness was the most common adverse event and showed a clear dose-dependence.

**Conclusions:**

This study demonstrated the safety and tolerability of neramexane treatment in patients with moderate to severe tinnitus. The primary efficacy variable showed a trend towards improvement of tinnitus suffering in the medium- and high-dose neramexane groups. This finding is in line with consistent beneficial effects observed in secondary assessment variables. These results allow appropriate dose selection for further studies.

**Trial Registration:**

ClinicalTrials.gov NCT00405886

## Background

Subjective tinnitus is commonly referred to as any sound experienced by a patient without recognizable source. It can be heard by most persons in absolute quietness. Intermittent tinnitus not in quietness, for periods of minutes or even hours, is also very common, occurring in about 10% of the population. Even persistent chronic tinnitus is not uncommon, in particular in elderly and hearing-handicapped individuals. A large-scale survey of the *Deutsche Tinnitusliga *(German Tinnitus Society) conducted in 1998 revealed that 4% of the German population suffers from tinnitus [1]. Up to 36% of patients in this survey additionally reported somatoform symptoms such as sleeping disturbances, depression and others, making tinnitus a severe burden for their daily life.

Although the pathophysiology of subjective tinnitus is poorly understood, and a definitive pathogenesis is unknown, both acetylcholine and *N*-methyl-D-aspartate (NMDA) receptors are thought to play important roles in the development of tinnitus. Disturbances of glutamatergic transmission have been implicated in various disorders of the central nervous system (CNS) and also in tinnitus [2]. These alterations may not only change the balance between excitatory and inhibitory brain processes in the auditory pathway, but are also likely to involve other brain structures. NMDA-receptor-relayed projections from the medial geniculate body to the lateral nucleus of the amygdala are of particular importance in a pathophysiological model of tinnitus in which connections between the auditory, limbic and autonomic nervous system have been suggested as playing a crucial part in the emergence of clinically relevant tinnitus [3]. The clinical observation that tinnitus sensation/perception - measured by pitch, maskability, and loudness - does not correlate with tinnitus severity in patients suffering from clinically important tinnitus [4-7] strongly supports the thesis that brain structures other than the auditory pathway must be involved.

In addition, NMDA receptor antagonists have been reported to afford protection from hearing loss caused by free-radical-induced damage to the hair cells [8].

The nicotinic acetylcholine receptor subunits α_9 _and α_10 _also play a relevant role in the efferent auditory system and the medial olivocochlear pathway [9,10]. Activation of α9α10 receptors inhibits mechanical amplification brought about by the activity of outer hair cells. As such, this nicotinic/cholinergic pathway could be an interesting target for pharmacological intervention in connection with impaired auditory processing [11,12]. In particular, patients suffering from chronic tinnitus and sensorineural hearing loss may have an imbalance in this system.

It is generally agreed that moderate-affinity, non-competitive NMDA receptor antagonists combine good efficacy and tolerability by preventing the pathological activation of NMDA receptors, but allowing their physiological activity [13]. Neramexane (1-amino-1,3,3,5,5-pentamethylcyclohexane) is a new compound with a dual mode of action, exhibiting antagonistic activity at the α_9_α_10 _nicotinic acetylcholine receptor [14] and the NMDA receptor [15]. Antagonistic properties at the 5-hydroytryptamin (5-HT_3_, serotonin) receptor [16] may also contribute to its mechanism of action in tinnitus. Neramexane was investigated for other indications in a number of Phase II/III studies. Overall, neramexane was safe and well tolerated in these studies.

So far, there are no well-established, specific medical treatments for tinnitus that provide replicable reduction of tinnitus and annoyance due to it [17]. The pharmacological effects of neramexane make it an interesting compound that might interfere in a positive manner with the pathophysiological processes involved in subjective tinnitus. Therefore we performed a randomized, double-blind, placebo-controlled clinical trial to assess the effect of neramexane in patients with moderate to severe tinnitus.

## Methods

This Phase II clinical trial was conducted at 37 centers in Austria and Germany as a randomized, placebo-controlled, double-blind, parallel-group, four-arm, dose-ranging study of neramexane in patients with moderate to severe subjective tinnitus. The study was compliant with the Declaration of Helsinki and was approved by Health Authorities and Ethics Committees and registered as NCT00405886 at ClinicalTrials.gov.

Patients 18 to 65 years of age at screening with a clinical diagnosis of persistent, subjective, moderate to severe, uni- or bilateral tinnitus present for at least 3 months but not more than 18 months were included. Diagnosis of these patients included a complete physical and ear, nose and throat examination and also included psychoacoustic measures and questionnaires including the Tinnitus Interview. In order to differentiate and characterize tinnitus patients in more detail, study recipients were subjected to puretone audiometry, tinnitus pitch and loudness matching and tinnitus masking. An auscultation around the ear and neck to check for vascular turbulences, inspection of the ear canal and the tympanic membrane, observation for nystagmus and tuning fork testing according to Weber and Rinne for unilateral, conductive, or sensorineural hearing loss were also performed.

Subjective tinnitus severity was graded using the 12-item German version (THI-12, German: TBF-12) of the 25-item English 'Tinnitus Handicap Inventory' (THI) patient questionnaire [18] and the physician's Clinical Global Impression of Tinnitus Severity (CGI-S) scale. Each question of the THI-12 can be answered by the patient with either 'often' (2points), 'sometimes' (1 point) or 'never' (0 points) with a maximum total score of 24 indicating most severe suffering from tinnitus. The CGI-S asks physicians to rate their patients' tinnitus severity based on their past experience with other patients with the same diagnosis and ranges from 1 (normal, not at all ill) to 7 (extremely ill). All patients included in the study were considered to have moderate to severe subjective tinnitus according to a THI-12 total score ≥9 and a CGI-S score of ≥4 at both screening and baseline. Patients had to have a body mass index (BMI) ≥18 kg/m^2 ^and ≤32 kg/m^2 ^and no clinically relevant abnormalities following physical examination and laboratory evaluation. Women of childbearing potential were required to practice adequate contraception. All patients provided written informed consent.

Patients were excluded if they presented with intermittent or pulsatile tinnitus or tinnitus as a concomitant symptom of another otological/neurological condition (e.g. otitis media). Patients with conductive hearing impairment were also excluded (air conduction threshold >20 dB worse than bone conduction threshold in at least two tested frequencies).

In addition, patients with epilepsy, acoustic neuroma, multiple sclerosis, serious head/cervical trauma with residual deficits, anamnestic HIV infection or any other clinically relevant neurological or psychiatric disorder or systemic disease (e.g. cardiac disease) following physical examination or assessment of medical history were excluded. Patients could not have received any other concomitant pharmacologic or non-pharmacologic treatment for tinnitus in the 28 days prior to screening (30 days if an investigational drug), including sound generators, counseling, behavioral therapy and psychotherapy. Pregnant and breastfeeding women were excluded.

**Figure 1:**
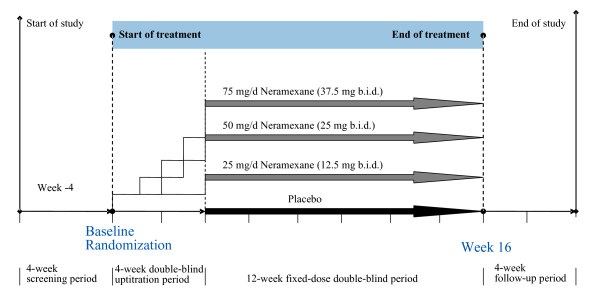
Study flow chart

Patients who met the inclusion criteria and none of the exclusion criteria were randomized to one of four treatment groups: neramexane 25 milligrams per day (mg/d), 50 mg/d or 75 mg/d or placebo. Treatment was administered over a 16-week period consisting of a 4-week up-titration period and a 12-week fixed-dose treatment period, with twice-daily dosing during both treatment periods. In cases where the trial drug in the 50 or 75 mg/d group was poorly tolerated, the investigator could consider a dose reduction by 25 mg/d. These cases received a reduced dosage throughout the trial, a subsequent rechallenge to the randomly assigned, higher dosage group was not allowed. After the treatment phase, administration of study medication was ceased immediately and patients were followed-up for further four weeks with no active treatment and with restrictions on concomitant therapy. In total, this study involved seven study visits: at screening, at baseline, and at the end of Weeks 4, 8, 12, 16, and 20 (Figure 1).

The primary efficacy endpoint was the absolute change in THI-12 total score from baseline to the endpoint visit (Visit 6, i.e. Week 16, or early termination). The THI-12 is derived from the 25 item version of the Tinnitus Handicap Inventory. The scale is easily administered and is a psychometrically robust and reliable tool to assess the different aspects of tinnitus suffering [18]. Although initially validated in German language only, it has meanwhile shown intercultural validation [19] which makes it a suitable tool for further international studies.

Secondary efficacy endpoints were the THI-12 total score (absolute values and change from baseline) at all further post-baseline visits, and the emotional-cognitive and the functional-communicational subscores of the THI-12 at all post-baseline visits. The results of a patient's self-assessment concerning tinnitus severity, tinnitus annoyance, tinnitus impact on life, tinnitus as a problem, hyperacusis as a problem, and hearing as a problem were assessed during a structured tinnitus interview and documented on 11-point Likert-like scales. Absolute values of the scores of these items at all visits as well as the changes from baseline were analyzed. Further criteria for assessment applied at all post-baseline visits were the interview-based clinical global impression of change (CGIC) and the total score and depression and anxiety subscores of the HADS (German version, HADS-D; [20]). The CGIC (item 27 of the tinnitus follow-up interview) was assessed by the patient according to a 7-item Likert scale ranging from 1 (very much improved or disappeared) to 7 (very much worse). As depression and anxiety are common co-morbidities in tinnitus patients, the HADS was used to monitor both conditions in the study population. The HADS is a reliable and validated questionnaire for non-psychiatrists to assess symptoms of depression and anxiety in an outpatient setting. In addition, the change in hearing-threshold levels (dB) of the left and right ears, the change in pitch match for the most troublesome tinnitus, the change in loudness match for the most troublesome tinnitus, and the change in minimum masking levels were determined.

Measures of safety, assessed at defined times, included standard clinical chemistry, coagulation, hematology and urinalysis, physical and ear, nose and throat examination, vital signs, 12-lead electrocardiography (ECG), urine drug screen and recording of spontaneously reported adverse events. An independent safety monitoring board continuously reviewed all serious adverse events and adverse events leading to discontinuation.

The confirmatory efficacy analysis was based on the intention-to-treat (ITT) analysis set, i.e. all patients who completed at least one post-baseline efficacy assessment for THI-12 total score and who received at least one dose of double-blinded study treatment. The change in THI-12 total score from baseline to the endpoint visit (i.e. Week 16 or early termination) was the primary efficacy endpoint in this study. Analysis of covariance (ANCOVA) with treatment group and center as factors and THI-12 baseline value as covariate was used for estimation of the treatment effects. The analysis was performed using the last-observation-carried-forward (LOCF) approach. Resulting *p*-values for pairwise treatment comparison (each active dose versus placebo) were evaluated for significance according to a hierarchical step-down testing procedure for dose groups in order to ensure an overall type I error of 5%. In addition, sensitivity analyses were performed on the "treated-per-protocol" population (TPP) and in an observed cases analysis.

Secondary efficacy criteria were analyzed similarly in an exploratory and descriptive manner (ITT and TPP). Incidence rates of treatment-emergent and serious adverse events, as well as events leading to discontinuation or dose reduction, were calculated; MedDRA coding for adverse events was used. Vital signs, ECG data and laboratory variables were analyzed by using descriptive statistics and were screened for potentially clinically significant values.

Patients were assigned to treatment groups in this multi-center study according to a balanced randomization procedure, using the RANCODE program (Version 3.6, IDV; Gauting, Germany). The randomization schedule and related relevant forms were kept sealed and locked in the Department of Total Quality Management (TQM) at Merz Pharmaceuticals GmbH and were not accessible until termination of the study.

The study was conducted in a double-blind fashion. The placebo tablets had the same appearance as neramexane tablets. Neither the investigator, nor the other medical staff, nor any patient knew the identity of any individual study medication. All other persons involved in the project (e.g. biostatisticians, data managers, monitors) were maintained blinded throughout the study. Members of the independent safety monitoring board were unblinded where this was necessary for them to perform a full case assessment.

## Results

**Figure 2:**
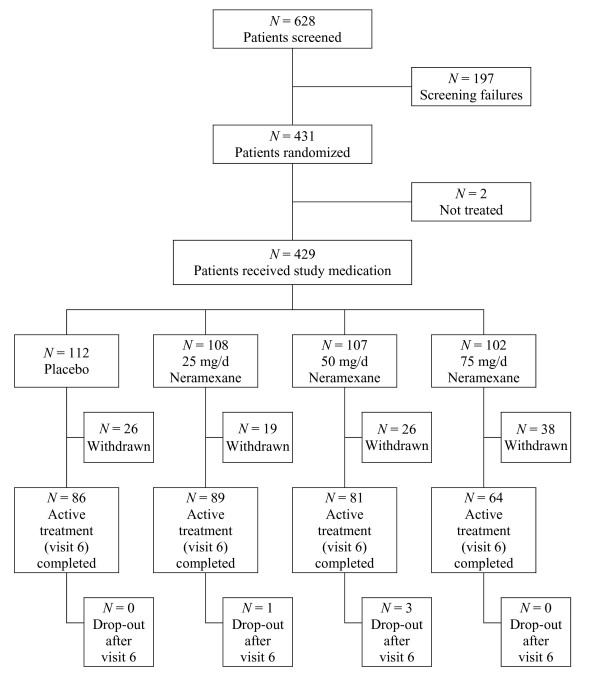
Patient disposition, flow chart

Patient disposition is summarized in Figure 2. Overall, 628 patients were screened from October 2005 to March 2007, although 197 of these patients were screened but not enrolled. The main reasons for screening failure included abnormal ECG, abnormal liver function, THI-12 score <9 and treatment with other concomitant medication (e.g. benzodiazepines). In total, 431 patients were randomized to study treatment; 429 patients received double-blind treatment and were included in the safety population (25 mg/d neramexane group, 108; 50 mg/d group, 107; 75 mg/d group, 102; placebo group, 112 patients). Two patients were randomized but did not receive study treatment. A total of 320 patients (74.6%) completed the study: 86 (77%) in the placebo group, 89 (82%) in the 25 mg/d group, 81 (76%) in the 50 mg/d group and 64 (63%) in the 75 mg/d group. The most frequent reason for discontinuation in all treatment groups was adverse events (13%, 8%, 22% and 28% for the placebo, 25 mg/d, 50 mg/d, and 75 mg/d neramexane group, respectively). Of the 431 randomized patients, 61 were excluded from the TPP population due to major protocol violations, mainly owing to their premature termination of study medication intake or other non-compliance.

Baseline demographic and anamnestic data were comparable among the treatment groups (Table 1). More than two thirds of patients in all treatment groups were male, Caucasian and non-smokers. Most patients had no or at most mild hearing loss. THI-12 total score at baseline was similar between all treatment groups. Audiometric and psychoacoustic outcomes for patients at baseline are shown in Table 2. The treatment groups were comparable in terms of puretone thresholds, tinnitus pitch and loudness matching and minimum masking level.

**Table 1:** Demographic and clinical characteristics at baseline (ITT population)

	Placebo		25 mg/d Neramexane		50 mg/d Neramexane		75 mg/d Neramexane	
	*N *= 111		*N *= 106		*N *= 106		*N *= 99	
	**Mean ± SD**		**Mean ± SD**		**Mean ± SD**		**Mean ± SD**	

Age (years)	45.7 ± 12.2		45.7 ± 11.8		44.9 ± 12.1		46.2 ± 11.8	
Height (m)	1.75 ± 0.10		1.77 ± 0.10		1.75 ± 0.10		1.76 ± 0.09	
Weight (kg)	78.81 ± 13.64		80.14 ± 14.16		77.23 ± 13.55		78.45 ± 12.73	
BMI (kg/m^2^)	25.75 ± 3.33		25.48 ± 3.06		25.16 ± 3.21		25.29 ± 3.04	
Mean tinnitus duration (months)	9.4 ± 4.64		9.5 ± 4.65		9.1 ± 4.17		8.8 ± 4.08	
THI-12 at baseline	14.4 ± 3.7		14.4 ± 3.9		14.5 ± 3.3		13.9 ± 3.7	

	***n***	**%**	***n***	**%**	***n***	**%**	***n***	**%**

Sex								
Male	76	68.5	75	70.8	72	67.9	68	68.7
Female	35	31.5	31	29.2	34	32.1	31	31.3
Ethnic origin								
Caucasian	109	98.2	105	99.1	105	99.1	98	99.0
Asian	0	0.0	0	0.0	1	0.9	1	1.0
Oriental	1	0.9	1	0.9	0	0.0	0	0.0
Other	1	0.9	0	0.0	0	0.0	0	0.0
Smoking habit								
Non-smoker	66	59.5	61	57.5	67	63.2	68	68.7
Smoker	27	24.3	22	20.8	19	17.9	17	17.2
Ex-smoker	18	16.2	23	21.7	20	18.9	14	14.1
Hearing loss (hearing threshold level)								
None (<20 dB)	64	57.7	65	61.3	61	57.5	61	61.6
Mild (20-40 dB)	42	37.8	33	31,1	43	40.6	30	30.3
Moderate (>40-70 dB)	5	4.5	8	7.5	1	0.9	5	5.1
Severe (>70-95 dB)	0	0.0	0	0.0	1	0.9	3	3.0

ITT = intent-to-treat, *N *= number of patients in specified group, *n *= number of patients,

SD = standard deviation

Calculation of percentages based on *N*.

**Table 2:** Audiometric and psychoacoustic characteristics of patients at screening (ITT population)

	*N*	Mean ± SD
**Puretone audiometry (worst ear)**		
Hearing threshold [dB]		
Placebo	111	19.9 ± 10.6
Neramexane 25 mg/d	106	20.1 ± 12.6
Neramexane 50 mg/d	106	19.4 ± 10.6
Neramexane 75 mg/d	99	20.6 ± 14.7
High frequency hearing threshold [dB]		
Placebo	111	36.9 ± 20.2
Neramexane 25 mg/d	106	36.0 ± 21.4
Neramexane 50 mg/d	106	36.6 ± 21.7
Neramexane 75 mg/d	99	36.9 ± 19.9
**Tinnitus matching (worst ear)**		
Frequency (pitch) match [Hz]		
Placebo	109	4577.5 ± 2601.9
Neramexane 25 mg/d	105	4915.1 ± 2596.2
Neramexane 50 mg/d	103	4958.7 ± 2683.1
Neramexane 75 mg/d	97	4920.9 ± 2804.8
Loudness match [dB]		
Placebo	105	38.6 ± 19.3
Neramexane 25 mg/d	103	40.5 ± 20.5
Neramexane 50 mg/d	99	39.3 ± 20.3
Neramexane 75 mg/d	91	39.2 ± 18.5
**Minimal masking level (worst ear)**		
Masking level [dB]		
Placebo	106	44.8 ± 21.0
Neramexane 25 mg/d	100	46.8 ± 19.0
Neramexane 50 mg/d	99	44.8 ± 21.1
Neramexane 75 mg/d	92	44.2 ± 20.6

ITT = intent-to-treat, *N *= number of patients, SD = standard deviation

**Figure 3:**
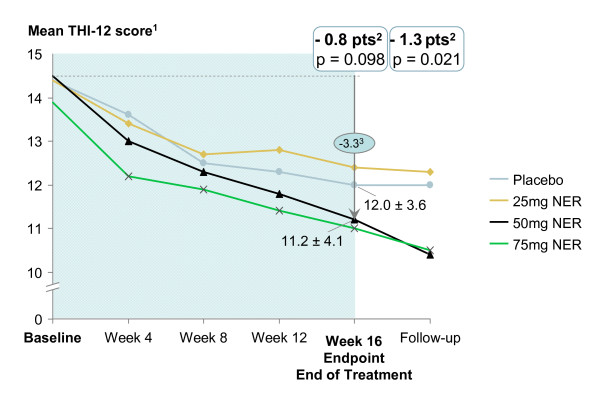
**Mean course of the primary efficacy variable (THI-12 score)** 1. Intention-to-treat, last observation carried forward. 2. Difference of least square means 50 mg vs. placebo  and p-values from ANCOVA with treatment and center as factor, baseline as covariate 3. Difference from  baseline, * p < 0.05

Figure 3 shows the course of the primary efficacy variable from screening to Week 20. Reductions in the THI-12 total score were observed in all treatment arms, but were highest in the medium- and high-dose neramexane groups. Comparison of changes between active groups and placebo yielded no statistical significance at the pre-defined endpoint, but a trend towards superiority for neramexane 50 mg/d and 75 mg/d (estimated treatment difference of 0.8 score points for 50 mg/d neramexane, *p *= 0.098, and 0.5 score points for 75 mg/d neramexane, *p *= 0.289). The 25 mg/d neramexane group was not superior to placebo.

During the follow-up period, the effects increased further for patients treated with 50 mg/d and 75 mg/d neramexane and, despite decreasing sample size, a *p*-value of 0.021 was found for the 50 mg group. The results of sensitivity analyses investigating the ITT-OC and the TPP population were consistent with the primary analysis (largest improvements for neramexane 50 mg/d group, estimated treatment differences 1.0 and 0.7 score points, respectively).

Table 3 summarizes the results of the main secondary variables throughout the trial. Statistically superior results of the patients treated with 50 mg/d neramexane compared with placebo-treated patients were found in the functional-communicational score of the THI-12. *P*-values below 0.05 were also shown for two items of the tinnitus interview, i.e. tinnitus annoyance and impact on life on an 11-point scale. Remaining variables consistently showed numerical superiority in the neramexane 50 mg/d group. No relevant changes were observed in either treatment group for puretone threshold, tinnitus pitch, loudness match, or minimum masking levels.

**Table 3:** Summary of main secondary variables at week 16 (end of treatment); intention to treat and LOCF analysis.

Variable (range)		Placebo (*N *= 111)	25 mg/d Neramexane (*N *= 106)	50 mg/d Neramexane (*N *= 106)	75 mg/d Neramexane (*N *= 99)
THI-12 Emotional-cognitive subscore	(0-14)	-1.7 ± 2.45	-1.2 ± 2.50 0.145	-2.0 ± 2.64 0.388	-1.8 ± 2.59 0.645

THI-12 Functional-communicational subscore	(0-10)	-0.7 ± 1.75	-0.7 ± 1.47 0.859	-1.2 ± 1.96 0.021*	-1.1 ± 1.77 0.097

Tinnitus loudness/severity	(0-10)	-0.7 ± 1.64	-0.4 ± 1.51 0.279	-0.9 ± 2.05 0.212	-0.9 ± 1.56 0.233

Tinnitus annoyance	(0-10)	-0.8 ± 2.04	-0.8 ± 1.95 0.639	-1.3 ± 2.26 0.043*	-0.8 ± 1.85 0.494

Tinnitus impact on life	(0-10)	-1.0 ± 1.98	-0.6 ± 1.89 0.335	-1.4 ± 2.61 0.038*	-0.8 ± 2.04 0.924

Tinnitus as a problem	(0-10)	-1.1 ± 1.86	-0.6 ± 1.72 0.058	-1.3 ± 2.42 0.328	-0.9 ± 2.05 0.951

Hyperacusis as a problem	(0-10)	-0.7 ± 2.51	-0.5 ± 2.49 0.271	-0.8 ± 2.72 0.778	-0.8 ± 1.85 0.666

Hearing as a problem	(0-10)	-0.2 ± 1.65	-0.0 ± 2.01 0.782	-0.7 ± 1.89 0.088	-0.1 ± 1.86 0.863

CGIC % improvement		27.9	22.6	34.0	38.4

Mean ± SD and *p*-values for changes, * *p *< 0.05 favoring treatment

(ANCOVA with treatment and center as factor, baseline as covariate).

Negative values indicate improvements.

No relevant change of the HADS score was observed. Decreases in the depression score (means ranging from -1.6 for placebo to -2.4 for the 50 mg/d group) were small and did not show a clear dose-dependency. This supports the hypotheses that neramexane specifically improves tinnitus symptoms and does not act through an antidepressant or anxiolytic effect. Patients without symptoms of anxiety or depression (HADS anxiety or HADS depression subscore < 10) benefited from neramexane treatment, whereas subjects with symptoms of anxiety or depression did not benefit. The estimated treatment difference for patients in the 50 mg dose group with a baseline depression subscore < 10 was 1.2 score points on the THI-12 (p = 0.022) and 1.3 score points (p = 0.030) for those patients with a baseline anxiety subscore < 10.

The most frequent adverse events are summarized in Table 4. The overall percentages of patients reporting at least one treatment-emergent adverse event, whether related to the study medication or not, were almost comparable between treatment groups. The lowest rate of adverse events (AEs) was observed in the 25 mg/d neramexane group (59.3%) followed by the placebo group (70.5%), the 50 mg/d neramexane group (73.8%) and finally the 75 mg/d neramexane group (78.4%).

**Table 4:** Frequency of patients with treatment-emergent adverse events by preferred term (safety population), incidence >5% in any treatment group

Preferred term (MedDRA 9.1)	Placebo (*N *= 112)		25 mg/d Neramexane (*N *= 108)		50 mg/d Neramexane (*N *= 107)		75 mg/d Neramexane (*N *= 102)	
	*n*	%	*n*	%	*n*	%	*n*	%
Any adverse event	79	70.5	64	59.3	79	73.8	80	78.4
Dizziness	9	8.0	11	10.2	21	19.6	38	37.3
Headache	15	13.4	11	10.2	14	13.1	11	10.8
Vertigo	1	0.9	3	2.8	10	9.3	11	10.8
Fatigue	3	2.7	4	3.7	9	8.4	8	7.8
Hypertension	3	2.7	1	0.9	3	2.8	6	5.9
Nasopharyngitis	9	8.0	7	6.5	6	5.6	6	5.9
Nausea	5	4.5	7	6.5	5	4.7	5	4.9

The percentages of patients who reported dizziness were similar in the placebo (8.0%) and 25 mg/d neramexane groups (10.2%) but increased with higher neramexane doses up to 37.3% in the 75 mg/d neramexane group. The incidence of vertigo also increased dose-dependently from the placebo to the high-dose neramexane group. The incidence of fatigue was higher in the 50 mg/d and 75 mg/d neramexane groups than in the other two treatment groups, but no clear dose-dependence was evident.

As shown in Table 5, the percentage of patients with treatment-emergent adverse events leading to dose reduction increased with neramexane dose. Dizziness, vertigo, nausea and fatigue were the most common treated-emergent adverse events leading to dose reduction.

**Table 5:** Frequency of treatment-emergent adverse events leading to dose reduction in more than one patient in any treatment group (safety population)

	Placebo (*N *= 112)		25 mg/d Neramexane (*N *= 108)		50 mg/d Neramexane (*N *= 107)		75 mg/d Neramexane (*N *= 102)	
	*n*	%	*n*	%	*n*	%	*n*	%
Any adverse event	4	3.6	7	6.5	9	8.4	21	20.6
Dizziness	1	0.9	2	1.9	7	6.5	15	14.7
Vertigo	0	0.0	1	0.9	2	1.9	5	4.9
Nausea	0	0.0	0	0.0	0	0.0	2	2.0
Fatigue	0	0.0	0	0.0	0	0.0	3	2.9

There was no relevant influence of the study medication on laboratory values, ECG or vital signs. No clinically meaningful changes in hematology, clinical chemistry or coagulation values were apparent from screening to week 8 or week 16 (or early termination) in any of the treatment groups. For the vast majority of patients, the ECG was assessed as normal. Furthermore, none of the abnormalities assessed as clinically relevant by the investigator fulfilled the criteria for potentially clinically significant PR, QRS or QTcB intervals. Mean and median values of blood pressure, pulse rate and weight were similar across all treatment groups and were stable throughout the study.

## Discussion

The primary objective of this study was to compare the efficacy, tolerability and safety of three different neramexane mesylate dosages (25, 50 and 75 mg/d) with placebo in patients with subjective tinnitus after a 16-week double-blind treatment period. Although statistically significant differences on the THI-12 total scale in Week 16 were not shown for any of the neramexane groups as compared with placebo, the study results nevertheless highlight the need for further investigation of neramexane as a possible treatment for patients with moderate to severe tinnitus.

During the initial 8 weeks of treatment, all patients, irrespective of their treatment, showed a distinct improvement of their tinnitus as measured by the THI-12 score. Thereafter, the THI-12 score further decreased solely in patients treated with the higher dosages of neramexane (50 and 75 mg/d), whereas placebo-treated patients and patients receiving the low dosage of neramexane (25 mg/d) showed no further improvement. It seems that the placebo effect abates or even ends after 8 weeks.

An interesting aspect of the trial is that, even after discontinuation of treatment, further improvement of the tinnitus symptoms occurred, resulting in statistically significant treatment differences for the neramexane 50 mg/d group in Week 20, which was 4 weeks after termination of the study medication (Figure 3). As the elimination half-life of neramexane is 30-45 hours, this further improvement is very unlikely to be attributable to a direct effect of the substance. As only one post-treatment measurement was performed in this study, this post-treatment effect is further investigated in an ongoing Phase 3 trial.

Measuring a purely subjective symptom is always a difficult clinical problem. Although the tinnitus questionnaire used is regarded as accurate and reliable [18] it is of great importance that further secondary results, measured in different ways, show consistent results. The measurement of tinnitus annoyance and tinnitus' impact on life by numerical or visual analog scales are commonly used instruments to assess tinnitus, and are recommended e.g. in the guidelines of the German Tinnitus Society [21].

There was no separation of dose groups in the audiometric and psychoacoustic measurements, including tinnitus masking and matching of frequency and loudness. This finding is consistent with publications confirming that the tinnitus suffering as perceived is not systematically dependent on audiometrically derived measures of tinnitus loudness and pitch [4-7]. A large number of patients with chronic and sub-chronic tinnitus are not bothered by its appearance. The disease is not characterized by the loudness of the sound but the inability of a patient to cope with this sound [22]. As such, validated Patient-Reported Outcome (PRO) questionnaires are more useful in assessing the suffering or nuisance level caused, and the patient's ability to cope with chronic tinnitus [23].

Neramexane was well tolerated, and no new safety concerns were raised by any of the observations made in this study. Neither laboratory values, nor ECG data, nor vital signs showed any important influence of the study medication. Overall, the incidence of adverse events (mainly dizziness, vertigo, and fatigue) increased with higher neramexane doses. Frequencies of AEs other than the three mentioned above showed no dose-relationship. Dizziness was a main reason for premature discontinuation in the medium- and high-dose group.

A placebo-controlled crossover study with memantine, an NMDA antagonist with moderate affinity, did not show a significant difference between memantine and placebo treatment on tinnitus suffering as assessed with the 25-item version of the THI [24]. However, as the authors conclude, the study had some methodological weaknesses (dose limitation, duration of wash-out phase) and the negative results do not generally exclude the usefulness of substances with NMDA antagonistic properties for the treatment of tinnitus.

## Conclusions

This proof-of-concept and dose-finding study demonstrated the safety and tolerability of neramexane treatment in patients with moderate to severe tinnitus receiving no concomitant tinnitus therapy. On the basis of the study results, the 50 mg/d neramexane mesylate dose is concluded to be the appropriate standard dose for further clinical development. In order to confirm this hypothesis, an international Phase III clinical trial program has now been started.

## Competing interests

MA, BEL, AG, RG, TR and HR, are employees and HJM, YW and HK are former employees of Merz Pharmaceuticals, which sponsored this clinical trial. MS, the co-ordinating investigator of this trial, received investigator fees and PJ has received lecturing fees from Merz Pharmaceuticals. **T**here are no other potential or actual competing financial interests.

## Authors' contributions

MS (Study design, evaluation and drafting of the manuscript), MA (Interpretation of data and drafting of the manuscript), BEL (Statistical analyses and drafting of the manuscript), AG (Study concept, and critical review of the manuscript), RG (Study design, evaluation and drafting of the manuscript), PJ (Methodological advice, evaluation and critical review of the manuscript), HJM (Study concept and critical review of the manuscript), TR (Interpretation of data and critical review of the manuscript), HR (Interpretation of data and critical review of the manuscript), YW (Statistical planning and drafting of the manuscript) and HK (Study design, interpretation of data and critical review of the manuscript).

All authors read and approved the final manuscript.

## Pre-publication history

The pre-publication history for this paper can be accessed here:

http://www.biomedcentral.com/1472-6815/11/1/prepub

## References

[B1] Pilgram M Rychlik R Lebisch H Siedentop H Goebel G Kirchhoff D Tinnitus in der Bundesrepublik Deutschland - eine repräsentative epidemiologische Studie HNO aktuell 1999 7 261 265

[B2] Eggermont JJ Tinnitus: neurobiological substrates Drug Discov Today 2005 10 1283 1290 10.1016/S1359-6446(05)03542-7 16214672

[B3] Lockwood AH Salvi RJ Coad ML Towsley ML Wack DS Murphy BW The functional neuroanatomy of tinnitus: evidence for limbic system links and neural plasticity Neurology 1998 50 114 120 9443467 10.1212/wnl.50.1.114

[B4] Folmer RL Griest SE Meikle MB Martin WH Tinnitus severity, loudness, and depression Otolaryngol Head Neck Surg 1999 121 48 51 10.1016/S0194-5998(99)70123-3 10388877

[B5] Henry JA Meikle MB Psychoacoustic measures of tinnitus J Am Acad Audiol 2000 11 138 155 10755810

[B6] Meikle MB Vernon J Johnson RM The perceived severity of tinnitus; some observations concerning a large population of tinnitus clinic patients Otolaryngol Head Neck Surg 1984 92 689 696 6440089 10.1177/019459988409200617

[B7] Hiller W Goebel G When tinnitus loudness and annoyance are discrepant: audiological characteristics and psychological profile Audiol Neurootol 2007 12 391 400 10.1159/000106482 17664870

[B8] Basile AS Huang JM Xie C Webster D Berlin C Skolnick P *N*-methyl-D-aspartate antagonists limit aminoglycoside antibiotic-induced hearing loss Nat Med 1996 2 1338 1343 10.1038/nm1296-1338 8946832

[B9] Elgoyhen AB Vetter DE Katz E Rothlin CV Heinemann SF Boulter J alpha10: a determinant of nicotinic cholinergic receptor function in mammalian vestibular and cochlear mechanosensory hair cells Proc Natl Acad Sci USA 2001 98 3501 3506 10.1073/pnas.051622798 11248107 PMC30682

[B10] Elgoyhen AB Johnson DS Boulter J Vetter DE Heinemann S Alpha 9: an acetylcholine receptor with novel pharmacological properties expressed in rat cochlear hair cells Cell 1994 79 705 715 10.1016/0092-8674(94)90555-X 7954834

[B11] Elgoyhen AB Katz E Fuchs PA The nicotinic receptor of cochlear hair cells: a possible pharmacotherapeutic target? Biochem Pharmacol 2009 78 712 719 10.1016/j.bcp.2009.05.023 19481062 PMC2737545

[B12] Veuillet E Bazin F Collet L Objective evidence of peripheral auditory disorders in learning-impaired children J Audiol Med 1999 8 18 29

[B13] Parsons CG Danysz W Bartmann A Spielmanns P Frankiewicz T Hesselink M Eilbacher B Quack G Amino-alkyl-cyclohexanes are novel uncompetitive NMDA receptor antagonists with strong voltage-dependency and fast blocking kinetics: in vitro and in vivo characterization Neuropharmacology 1999 38 85 108 10.1016/S0028-3908(98)00161-0 10193901

[B14] Plazas PV Savino J Kracun S Gomez-Casati ME Katz E Parsons CG Millar NS Elgoyhen AB Inhibition of the alpha9alpha10 nicotinic cholinergic receptor by Neramexane, an open channel blocker of N methyl-D aspartate receptors Eur J Pharmacol 2007 566 11 19 10.1016/j.ejphar.2007.03.026 17466293

[B15] Danysz W Parsons CG Jirgensons A Kauss V Tillner J Amino-alkyl-cyclohexanes as a novel class of uncompetitive NMDA receptor antagonists Curr Pharm Des 2002 8 835 843 10.2174/1381612024607117 11945134

[B16] Rammes G Rupprecht R Ferrari U Zieglgänsberger W Parsons CG The *N*-methyl-D-aspartate receptor channel blockers memantine, MRZ 2/579 and other amino-alkyl-cyclohexanes antagonise 5-HT(3) receptor currents in cultured HEK-293 and N1E-115 cell systems in a non-competitive manner Neurosci Lett 2001 306 81 84 10.1016/S0304-3940(01)01872-9 11403963

[B17] Noble W Treatments for tinnitus Trends Amplif 2008 12 236 241 10.1177/1084713808320552 18635586 PMC4134891

[B18] Greimel KV Leibetseder M Unterrainer J Biesinger E Albegger K Der Tinnitus-Beeinträchtigungs-Fragebogen (TBF-12). Übersetzung und Adaption Verhaltenstherapie und Verhaltensmedizin 2000 21 39 49

[B19] Korbel US Görtelmeyer R Elkin E Intercultural validation of the Tinnitus Handicap Inventory 12 (THI-12) 4th Tinnitus Research Initiative Meeting 2010 Dallas, Texas

[B20] Zigmond AS Snaith RP The hospital anxiety and depression scale Acta Psychiatr Scand 1983 67 361 370 10.1111/j.1600-0447.1983.tb09716.x 6880820

[B21] Biesinger E Hesse G Mazurek B Zenner HP Brusis T Goebel G Kröner-Herwig B Michel O Arnold W AWMF online: Leitlinien der Deutschen Gesellschaft für Hals-Nasen-Ohren-Heilkunde, Kopf- und Hals-Chirurgie: Tinnitus http://www.uni-duesseldorf.de/AWMF/ll/017-064.htm

[B22] Budd RJ Pugh R Tinnitus coping style and its relationship to tinnitus severity and emotional distress J Psychosomatic Res 1996 41 327 335 10.1016/S0022-3999(96)00171-7 8971662

[B23] Langguth B, Goodey R, Azevedo A, Bjorne A, Cacace A, Crocetti A, Del Bo L, De Ridder D, Diges I, Elbert T, Flor H, Herraiz C, Ganz Sanchez T, Eichhammer P, Figueiredo R, Hajak G, Kleinjung T, Landgrebe M, Londero A, Lainez MJ, Mazzoli M, Meikle MB, Melcher J, Rauschecker JP, Sand PG, Struve M, Van de Heyning P, Van Dijk P, Vergara R Consensus for tinnitus patient assessment and treatment outcome measurement Tinnitus Research Initiative Meeting: XX-YY July 2006; Regensburg, Germany. Prog Brain Res 2007 166 525 36 10.1016/S0079-6123(07)66050-6 PMC4283806 17956816

[B24] Figueiredo RR Langguth B Mello de Oliveira P Aparecida de Azevedo A Tinnitus treatment with memantine Otolaryngol Head Neck Surg 2008 138 492 496 10.1016/j.otohns.2007.11.027 18359360

